# Effects of a Footbathing Intervention on Physiological, Endocrine, and Psychological Status in Japanese University Students: A Randomized Controlled Trial

**DOI:** 10.3390/ijerph22010022

**Published:** 2024-12-28

**Authors:** Kaho Yamasaki, Hiromitsu Miyata

**Affiliations:** 1 Graduate School of Letters, Arts and Sciences, Waseda University, Tokyo 162-8644, Japan; 2 Faculty of Letters, Arts and Sciences, Waseda University, Tokyo 162-8644, Japan; miyata@waseda.jp

**Keywords:** footbathing, interoceptive awareness, health, cortisol, autonomic nervous system activities

## Abstract

The present pilot study examined effectiveness of a 2-week footbathing intervention on physiological, endocrine, and psychological status in healthy Japanese university students. A total of 51 participants were randomly assigned to a footbathing or normal bathing group. Participants in both groups provided daily free descriptions of their physical and mental states during the intervention period. Participants also underwent measurements of autonomic nervous system activities and salivary cortisol, and completed questionnaires in the pre- and post-intervention periods, as well as in the follow-up period. Neither the footbathing group nor the normal bathing group showed significant changes in deep body temperature, blood pressure, or salivary cortisol through the intervention period. Significant increases in dispositional mindfulness and interoceptive awareness, and significant decrease in trait anxiety were observed regardless of the groups. Nevertheless, an awareness of changes in bodily sensations and mood by footbathing mentioned in the participants’ free descriptions was significantly associated with increased deep body temperature, dispositional mindfulness, interoceptive awareness, and subjective well-being from the pre- to post-intervention periods. These results suggest that the period of intervention and sample size might have been insufficient to induce significant changes in baseline psychophysiological status, but that awareness of changes in psychophysiological states may potentially be involved in the mechanism of footbathing.

## 1. Introduction

Bathing techniques have been traditional regimens practiced in the East since ancient times [1]. Among them, footbathing is a convenient method involving the immersion of feet in hot water, typically covering from the toes to the ankles or legs. Due to its simplicity, footbathing can easily be incorporated into daily life [2], and is widely used as a form of thermotherapy [3,4]. Footbathing is considered to significantly contribute to physical and mental health by promoting blood circulation and warming the body [1,5].

There is growing evidence suggesting that footbathing has various health benefits [6]. For example, footbathing is known to enhance immune functions [7], improve arterial health [2], and promote sleep onset and improve sleep quality [8,9]. Footbathing has also been shown to improve specific diseases or symptoms. Empirical studies have reported that footbathing contributed to alleviating menstrual pain [10], pregnancy-related lower back pain, and pelvic girdle pain [11]. Furthermore, footbathing has been shown to alter subjective evaluations of psychological traits or states. Effati-Daryani et al. [12] conducted an 8-week intervention of footbathing with lavender cream, and reported significant reductions in stress, anxiety, and depression in pregnant women. Lee et al. [13] reported that an inter footbathing by using essential oils for 30 min significantly reduced stress and improved mood states in stroke patients. Mazloam et al. [14] and Rajabzadeh et al. [15] both reported that a two-week intervention of footbathing involving patients undergoing radiotherapy relieved fatigue or depression. These empirical studies suggest that footbathing may alleviate psychological symptoms resulting from specific physiological conditions.

These health benefits are hypothesized to be associated with changes in the autonomic nervous or endocrine activities induced by footbathing [16]. The physiological effects of footbathing have been shown in previous studies. For example, Saeki [17] introduced footbathing lasting for 10 min by using either regular or lavender oil, and examined their effects on autonomic nervous system activities in Japanese undergraduate students. Results showed an increase in the high frequency (HF) component of heart rate variability (HRV) five minutes after the start of footbathing, even with the regular footbathing. In another empirical study involving Chinese female university students with menstrual pain, individuals who performed a 20-min seated footbathing showed lower low frequency/high frequency (LF/HF) ratio of HRV at the end of footbathing compared to individuals in the control group. These data suggested enhanced relaxation resulting from decreased sympathetic nervous system activities [10]. Similarly, Yamamoto et al. [16] reported a decrease in the LF/HF ratio of HRV after a 20-min supine footbathing in healthy Japanese adult men. These empirical studies suggest that parasympathetic nervous system activities may be enhanced by footbathing interventions, which may contribute to an improved sympatho-vagal balance during or immediately after footbathing. Regarding endocrine activities, Yamamoto et al. [16] reported that plasma cortisol and secretory immunoglobulin A (s-IgA) levels declined during a 20-min footbathing compared to the baseline in healthy adult men. McCullough et al. [11] conducted a 6-week footbathing intervention in pregnant women and found an increase in salivary *β*-endorphin levels, which were significantly associated with pain relief, euphoria, and well-being. Based on these findings, it can be hypothesized that footbathing contributes to reducing physical and mental stress by regulating stress hormone secretion.

Another potential factor relevant to the process through which footbathing can enhance physical and mental health is heightened awareness of interoception, defined as the perception of internal bodily states [18,19]. Footbathing can be deemed as an interoceptive stimulus involving thermal and visceral stimulation because it is a thermotherapy that utilizes blood circulation. Some bidirectional transmissions of internal bodily state information via autonomic nervous systems rise to consciousness [20,21]. Interventions that provide interoceptive stimuli can repetitively alter the perception of interoception through regulation of autonomic nervous system activities [19]. Such salient interoceptive stimuli induce bottom-up attention to inner self when a salient event is detected [22]. Computational simulations suggest that directing attention towards interoception contributes to choosing appropriate actions to protect homeostasis through enhancing accuracy in awareness and predictions of moment-to-moment bodily states [21]. Conversely, it is known that failures in accurately predicting changes in interoceptive states can lead to depression and/or anxiety disorders [23]. Given these underpinnings, it seems reasonable to assume that an intervention by footbathing may contribute to enhancing physical and mental health via increased attention to/awareness of interoception.

Despite the developments in research, there is little evidence to date on changes in baseline autonomic/endocrine activities, psychological health induced by continued regular footbathing. Most preceding studies on footbathing typically involved a single-session footbathing in a laboratory setting, and measured physiological, endocrine, and/or psychological states associated with footbathing. In a few previous studies that involved several weeks of footbathing interventions [7,12,13,17], footbathing was often combined with other interventions such as aromatherapy or electrical stimulations, so that the standalone effects of footbathing remain unclear. In addition, whether footbathing induces increase in awareness of one’s own physical and mental states is yet to be examined. Furthermore, although many previous studies used HF as an indicator of parasympathetic nervous system activity and LF/HF as a marker of sympathovagal balance, reliability of these interpretations has been debated [24,25,26]. Therefore, involvement of more reliable physiological measures, such as blood pressure, should be required to better demonstrate the physiological effects of footbathing.

Given these research contexts, the present pilot study aimed to examine potential efficacy of footbathing on physiological, endocrine, and psychological health statuses in healthy Japanese university students by introducing a two-week intervention. We hypothesized that practice of footbathing contributes to increase in psychological health through enhanced homeostatic regulation at physiological and endocrine levels, as well as through heightened awareness of interoception. Specifically, we expected to observe physiological and endocrine changes such as an increase in deep body temperature and decrease in blood pressure and salivary cortisol levels from the pre- to the post-intervention period and follow-up. We also expected to observe changes in psychological status including increases in dispositional mindfulness, which encompasses focusing attention to the present experience without evaluation or judgment [27], interoceptive awareness, and subjective well-being, and decrease in trait anxiety and depression from the pre- to the post-intervention period and follow-up. In addition to these quantitative measures, we also analyzed qualitative data including the participants’ free descriptions of their self-awareness regarding physical and mental states during the two-week intervention period. We expected that participants who practiced footbathing would exhibit increased awareness of bodily sensations, especially those related to thermal effects, along with improvements in their physical condition and mood in their free descriptions.

## 2. Methods

### 2.1. Participants

**Figure 1:**
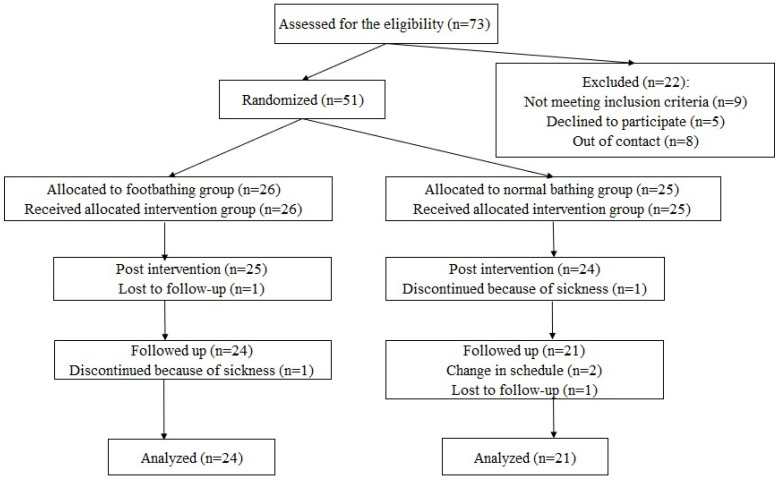
A flow diagram of participation.

Healthy Japanese undergraduate and graduate students participated in the study. A flow diagram of participation is shown in Figure 1. The sample size was determined in accordance with a report by Whitehead et al. [28]. Whitehead et al. optimized the required sample size for an internal pilot trial using the non-central *t*-distribution approach to minimize the sample size of the main trial. These calculations suggested that 44 participants were required for a pilot trial involving two treatment arms with a medium effect size (*d*= 0.50, significance level (α) = 0.05, power (1 − *β*) = 0.80). To account for potential dropouts, we recruited approximately 30 more participants than the required number. Participants were recruited through the university’s online system for posting part-time jobs. To exclude individuals with severe depressive tendencies for ethical considerations, potential participants were screened by using the Japanese version of the Center for Epidemiologic Studies Depression Scale (CES-D; [29]). Based on previous research, individuals whose total CES-D scores were the cutoff value (27 points) or above were excluded [30,31]. Individuals either having treatment at a psychiatric or psychosomatic clinic or participating in other intervention studies were also excluded to prevent potential interference of other treatments or interventions. Applicants aged 18–25 who did not meet the abovementioned exclusion criteria were included as participants in the study. When initially visiting the laboratory before starting the intervention, participants who met the inclusion criteria received an explanation of the purpose and procedure of the study through both written and verbal instructions. Specifically, participants were informed that the risks for health associated with footbathing was low, even though short-term headaches and/or a feeling of fatigue might occur as a result of lifestyle changes associated with the intervention. Participants were also instructed to quit the interventions and contact the researcher readily when they felt unwell during the intervention period. After the explanation, all participants provided written informed consent upon agreement to co-operate. There were a total of 51 participants (18 males, 33 females, mean age = 20.4 years, *SD* = 1.83, 69.9% of applicants), who were adaptively randomly assigned to either the footbathing group (9 males, 17 females, mean age = 20.2 years, *SD* = 1.82) or the normal bathing group (9 males, 16 females, mean age = 20.5 years, *SD* = 1.87; Figure 1). Distribution of gender and age was balanced across groups. Out of the 51 participants who started the intervention, 45 participants (88.2% of the participants) who completed the follow-up assessment were included in the analysis: 24 from the footbathing group (9 males and 15 females, mean age = 20.4 years, *SD* = 1.81) and 21 from the normal bathing group (7 males and 14 females, mean age = 20.5 years, *SD* = 2.04; Figure 1). The data collection was conducted between May 2022 and July 2023.

### 2.2. Intervention by Footbathing

Participants in the footbathing group were instructed to perform a 15-min footbathing every day for two weeks before bedtime. Specifically, participants were instructed to immerse their legs up to below the knees (covering the entire lower legs) in 42 to 43 °C water in their own home bathtub while seating. Participants in the normal bathing group continued their usual bathing routines during the same period without performing footbathing. To investigate qualitative changes in the psychosomatic states during the intervention period, participants from both groups were instructed to provide free descriptions on noticeable changes in their physical condition and mood, as well as anything they thought or felt each day before bedtime.

### 2.3. Procedure

All participants visited the laboratory located within the university three times: (1) immediately before starting the intervention (pre-intervention), (2) immediately after the intervention period was over (post-intervention), and (3) two weeks after the intervention ended (follow-up). During each visit, participants underwent measurements of autonomic nervous system activities, provided saliva samples, and completed questionnaires. All procedures were performed while participants were seated at rest, and each visit lasted approximately 30 min. To minimize the impact of diurnal variations in autonomic nervous system and endocrine activities, visiting hours were scheduled between 1:00 PM and 6:00 PM, and each participant visited the laboratory at the same time for all three visits. To avoid confounding environmental factors in autonomic nervous system activities and salivary stress hormone concentrations, participants were instructed to avoid strenuous physical activities, eating, drinking, or brushing their teeth within one hour prior to arrival at the laboratory.

### 2.4. Physiological and Endocrine Measures

***Autonomic Nervous System Activities.*** Deep body temperature (tympanic temperature) was measured by using an ear thermometer (MC-510 *Kenon-kun Mimi*; Omron, Kyoto, Japan). Additionally, systolic and diastolic blood pressure were measured within one minute using a wrist blood pressure monitor (EW-BW35; Panasonic, Tokyo, Japan). The blood pressure monitor was placed on the left wrist. Participants rested their elbow on a table to stabilize the position of the blood pressure monitor, ensuring that the measurement site was approximately at the same level as the heart. Blood pressure was measured twice consecutively, and the averages of these scores were used for analysis.

***Salivary Cortisol.*** To assess the baseline cortisol levels as a biomarker of physiological stress responses, a total of 0.5 mL of saliva was collected during each visit by using saliva collection instruments (Saliva Collection Aid/Cryovial; Salimetrics, Carlsbad, CA, USA). Immediately after collection of the salivary samples, they were frozen at −30 °C in a freezer located within the laboratory.

### 2.5. Psychological Scales

***Mindfulness.*** The present study used the Five Facet Mindfulness Questionnaire (FFMQ; [32]), a psychological scale commonly used to measure dispositional mindfulness. The FFMQ consists of 39 items across five subscales: *Observing* (8 items), *Describing* (8 items), *Acting with Awareness* (8 items), *Non-judging of inner experience* (8 items), and *Non-reactivity to inner experience* (7 items). Participants rated each item on a 5-point scale ranging from 1 (never or very rarely true) to 5 (very often or always true). The present study used the Japanese version of the FFMQ, which has been developed and validated by Sugiura et al. [33]. Previous studies conducted both in Japan and other countries have widely confirmed the validity of both the five-factor and one-factor structures of the FFMQ [32,33,34]. Scores for each subscale and the total score were calculated.

***Interoceptive awareness.*** The Multidimensional Assessment of Interoceptive Awareness (MAIA) is a widely used scale to evaluate degrees of interoceptive awareness [35]. The present study used the Japanese version of the MAIA, which was developed and validated by Shoji et al. [36]. The Japanese version of the MAIA is composed of 25 items that fall into either of the six subscales including *Attention regulation*, *Body listening*, *Noticing*, *Emotional awareness*, *Trusting*, and *Not-distracting*. Participants evaluated the extent to which each item described their daily experiences on a 6-point scale ranging from 0 (not at all) to 5 (always). Average scores of each subscale were used for analyses.

***Subjective well-being.*** The present study used the Subjective Well-Being Scale (SWBS), which was developed and validated by Ito et al. [37]. The SWBS is based on the Subjective Well-Being Inventory (SUBI) developed by the World Health Organization (WHO), which is a scale used to assess levels of psychological health and stress [38,39]. The SWBS comprises 15 items across five dimensions: *General well-being― positive affect, Confidence in coping, expectation-achievement―congruence*, *General well-being― negative affect*, and *Transcendence*. Participants rated each item on a 4-point scale ranging from 1 (*not at all*, *never*, etc.) to 4 (*very much*, *always*, etc.). The total score of all items was used for analyses.

***Trait anxiety.*** The State-Trait Anxiety Inventory (STAI), developed by Spielberger et al. [40], is one of the most widely used scales for measuring anxiety. The STAI consists of two scales: the State Anxiety Scale (STAI-S) and the Trait Anxiety Scale (STAI-T). The STAI-S measures temporary emotional states such as concerns and tension pertaining to specific events, while the STAI-T assesses susceptibility to experience state anxiety under stressful conditions, indicating a more stable anxiety disposition. We used the Japanese version of the STAI-T, which was developed by Shimizu and Imae [41]. Participants rated the extent to which each of the 20 items applies to their normal status on a 4-point scale ranging from 1 (*not at all*) to 4 (*always*). The total score of STAI-T was used for analyses.

***Depression.*** We used the Center for Epidemiologic Studies Depression Scale (CES-D), which was developed for clinical research on depression [42]. The Japanese version of CES-D [29] has shown clinical effectiveness in assessing depressive symptoms. The CES-D consists of 20 items that inquire about emotional and physical symptoms related to depression, such as mood, appetite, and sleep. Participants rated the frequency of experiencing each emotional and physical state over the past week on a 4-point scale from A to D, with each alphabet corresponding to rarely or none of the time (less than 1 day per week), some or a little of the time (1–2 days), occasionally or a moderate amount of time (3–4 days), and most or all of the time (5–7 days), respectively. After data collection, each alphabet was replaced by a number from 0 to 3, respectively. The total scores were obtained by summing up responses to all items, which were used for analyses.

### 2.6. Analysis of Cortisol Concentration

Centrifugation and analysis of cortisol concentration for the saliva samples were outsourced to Yanaihara Institute Inc., Fujinomiya, Japan. Freeze-preserved samples were sent to the institute in a styrofoam box with ice packs. The saliva samples had been centrifugated at 3000 rpm for 10 min and analyzed using the Enzyme-linked Immunosorbent Assay method (Salivary Cortisol EIA Kit; Salimetrics, Carlsbad, CA, USA). The analysis used 50 μL of saliva per determination. All samples were analyzed twice, and their average was used as representative values. Analysis of each participant’s sample across the three measurement periods was conducted by using the same plate. The average intra-assay coefficient of variation was 6.0%.

### 2.7. Statistical Analysis Methods

Statistical analyses for the physiological, endocrine, and psychological measures were conducted using SPSS Version 29.0.2.0. First, we calculated means and standard deviations (*SD*s) for each measure collected at the pre-intervention, post-intervention, and follow-up assessments, respectively. Subsequently, we conducted a two-way mixed-design analyses of variance (ANOVA) with group (2) as a between-participant factor and measurement period (3) as a within-participant factor, involving each measure as a dependent variable. When sphericity assumption was not valid in Mauchly’s test, Greenhouse–Geisser correction was applied for degrees of freedom [43]. For statistically significant interactions between the group and measurement period factors, simple main effect tests were conducted. Significant simple main effects were further analyzed by post-hoc *t*-tests with Bonferroni corrected alpha levels (0.05/3) for comparisons between measurement periods at specific groups, and without correction for comparisons between the groups at a specific measurement period. For variables indicating a significant main effect of the group factor and insignificant interactions, scores pooling all measurement periods were compared between the footbathing and normal bathing groups by a *t*-test. For variables indicating a significant main effect of the measurement period factor and insignificant interactions, multiple comparisons by *t*-tests with Bonferroni correction (α = 0.05/3) were conducted for scores pooled across groups at each period.

Next, we performed a quantitative text analysis of the participants’ free descriptions collected during the intervention period using KH Coder 3 [44]. Prior to the analysis, unnecessary symbols were removed as preprocessing of the data. A morphological analysis using MeCab identified a total of 23,168 words (2062 unique words), of which 9425 words (1711 unique words) were used for the analysis after excluding particles and auxiliary verbs. We then conducted a multiple correspondence analysis of the extracted words (top 60 words by Jaccard coefficients) and an external variable (group), to visualize characteristic words within the descriptions for the footbathing and normal bathing groups. Additionally, a co-occurrence network analysis was conducted using only the descriptions from the footbathing group (5563 words in total; 1101 unique words), to examine the relationships between words. We calculated the Jaccard coefficients between strongly related words and visualized the top 100 of them in the network. The modularity-based method was used to detect subgraphs of words to visualize words that were strongly related to each other [45].

In addition, we coded the content of the free descriptions by participants from the footbathing group and examined their associations with changes in quantitative variables from pre- to post-assessments. In the correspondence and co-occurrence network analyses, synonymous words were recognized as different words, even when expressing the same content, leading to dispersed frequencies. Moreover, sole multivariate analyses mechanically summarize the content without reflecting the analysts’ hypotheses or concerns [46]. Therefore, in the subsequent stages, we conducted coding to group and analyze expressions related to the practice of footbathing. Rules for coding were developed by extracting and classifying similar expressions from KH Coder’s list of extracted words, based on characteristic words and their relationships identified in the correspondence and co-occurrence network analyses. For each participant’s 2-week free descriptions from the footbathing group, we quantified the presence (1) or absence (0) of each code. Finally, we calculated Spearman’s rank correlation coefficients between the presence of specific codes and changes in scores from physiological, endocrine, and psychological measures from pre- to post-intervention assessments.

## 3. Results

### 3.1. Changes in Physiological, Endocrine, and Psychological Measures

Table 1 shows means and standard deviations for each physiological, endocrine, and psychological measure obtained at each measurement period for the footbathing and normal bathing groups. In addition, Table 2 shows results from two-way ANOVAs conducted for each measure. Interaction between group and measurement period was not statistically significant for all measures except for the CES-D. Regarding the CES-D, simple main effects of measurement period were significant in neither the footbathing group (*F* [2, 42] = 2.412, *p* = 0.102, *ηp*^2^ = 0.103) nor the normal bathing group (*F* [2, 42] = 2.595, *p* = .087, *ηp*^2^ = 0.110). Thus, even though patterns of changes in depression differed between the groups, no significant differences between specific groups or measurement periods were observed.

The main effects of measurement period were statistically significant for the total scores from the FFMQ and its two subscales, i.e., non-reactivity and non-judging, for the two subscales from the MAIA, i.e., body listening and trusting, and for the STAI-T. In multiple comparisons, the total scores from the FFMQ showed a significant increase both from pre- to post-intervention (*t*_44_ = 3.771, *p* = .034, corrected, 95%CI = [0.217, 7.325], *d* = 0.390) and from pre-intervention to follow-up (*t*_44_ = 5.259, *p* = .005, corrected, 95%CI = [1.327, 9.191], *d* = 0.374), but not from post to follow-up (*t*_44_ = 1.488, *p* = .816, corrected, 95%CI = [−1.843, 4.819], *d* = 0.109). Scores from the body listening subscale of the MAIA also significantly increased from pre- to post-intervention (*t*_44_ = 1.580, *p* = .049, corrected, 95%CI = [0.004, 3.156], *d* = 0.357) and from pre-intervention to follow-up (*t*_44_ = 1.524, *p* = .020, corrected, 95%CI = [0.188, 2.859], *d* = 0.356), but not from post to follow-up (*t*_44_ = −0.057, *p* = 1.000, corrected, 95%CI = [−1.308, 1.195], *d* = 0.009). Scores from the non-reactivity subscale from the FFMQ significantly increased from pre-intervention to follow-up (*t*_44_ = 1.520, *p* = .023, corrected, 95%CI = [0.138, 2.362], *d* = 0.352), but not from pre-intervention to post-intervention (*t*_44_ = 0.774, *p* = .121, corrected, 95%CI = [−0.137, 1.685], *d* = 0.220) or post-intervention to follow-up (*t*_44_ = 0.476, *p* = .666, corrected, 95%CI = [−0.481, 1.433], *d* = 0.140). Similarly, the trusting subscale from the MAIA also significantly increased from pre-intervention to follow-up (*t*_44_ = 1.616, *p* = .032, corrected, 95%CI = [0.110, 3.122], *d* = 0.438), but not from pre-intervention to post-intervention (*t*_44_ = 1.018, *p* = .158, corrected, 95%CI = [−0.255, 2.291], *d* = 0.277) or post-intervention to follow-up (*t*_44_ = 0.598, *p* = .265, corrected, 95%CI = [−0.257, 1.453], *d* = 0.241). The total scores from the STAI-T significantly decreased from pre-intervention to follow-up (*t*_44_ = −2.461, *p* = .025, corrected, 95% CI = [−4.681, 0.242], *d* = 0.246), but not from pre-intervention to post-intervention (*t*_44_ = −1.384, *p* = .175, corrected, 95% CI = [−3.157, 0.390], *d* = 0.147) or post-intervention to follow-up (*t*_44_ = −1.077, *p* = .165, corrected, 95% CI = [−2.438, 0.283], *d* = 0.106). On the other hand, the non-judging subscale scores from the FFMQ did not significantly change from pre-intervention to follow-up (*t*_44_ = 1.926, *p* = .051, corrected, 95% CI = [−0.005, 3.856], *d* = 0.321), pre-intervention to post-intervention (*t*_44_ = 1.473, *p* = .077, corrected, 95% CI = [−0.113, 3.059], *d* = 0.268) or post-intervention to follow-up (*t*_44_ = 0.452, *p* > .999, corrected, 95% CI = [−0.935, 1.840], *d* = 0.079). These data show that mindfulness and its multiple components as well as interoceptive awareness increased, and trait anxiety decreased across measurement periods regardless of the groups. The main effect of group was not statistically significant for any of the physiological, endocrine, or psychological measures.

### 3.2. Quantitative Text Analysis of Free Descriptions

**Figure 2:**
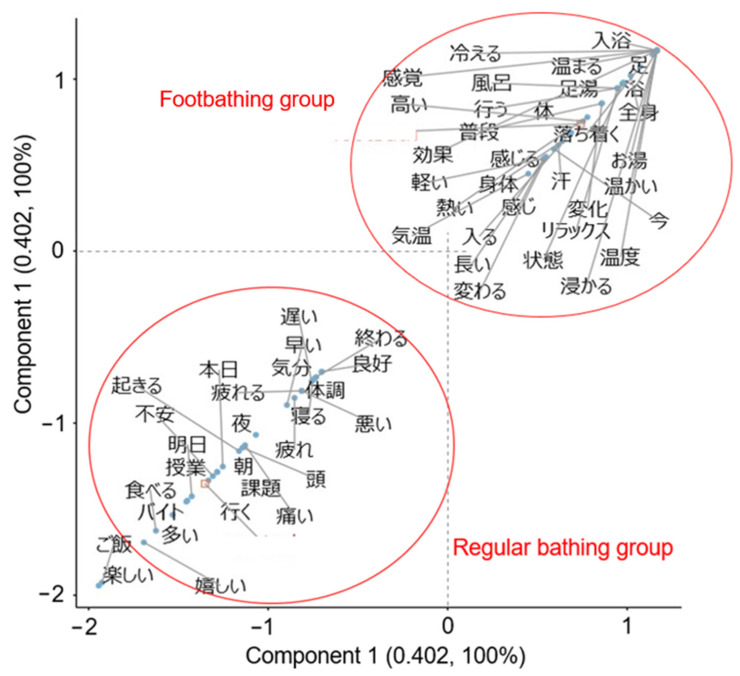
Results from a correspondence analysis involving group as an external variable. Japanese characters within the figure show original Japanese words extracted from the participants’ free descriptions, i.e., “温かい (warm),” “効果 (effect),” etc. See Table 3 and the main text for correspondence between each Japanese word and its English equivalent.

Participants in the footbathing group showed descriptions such as “My toes stayed warm until bedtime” “I felt fatigued, but my body felt slightly lighter” and “I feel a great relaxation by warming my feet before taking a full-body bath, and I want to continue doing this”. In the normal bathing group, descriptions such as “Today was tough with a lot of classes. I feel a bit down remembering the test next week” and “Feeling good because I had time for my hobbies such as listening to music and reading a book” were obtained. Results from a multiple correspondence analysis for extracted words and an external variable (group) are depicted in Figure 2. In the correspondence analysis plot, words that were commonly used, regardless of group, are displayed near the origin. On the other hand, words that were differentially extracted in each group are displayed in the same direction as the representations of each group name, and are located farther away from the origin. In the footbathing group, words pertaining to the practice and effects of footbathing, such as shintai “body”, “relax”, kouka “effects”, and atatakai “warm” were extracted as characteristic words. On the other hand, words related to daily life (e.g., kadai “task”, jugyo “lecture”, and baito “part-time job”) and emotional words (e.g., fuan “anxious”, ureshii “happy”, and tanoshii “enjoyable”) were extracted as words characterizing the normal bathing group. In the present samples, all characteristic words for each group appeared prominently in each group’s descriptions, and no words were found to be common between the groups.

**Figure 3:**
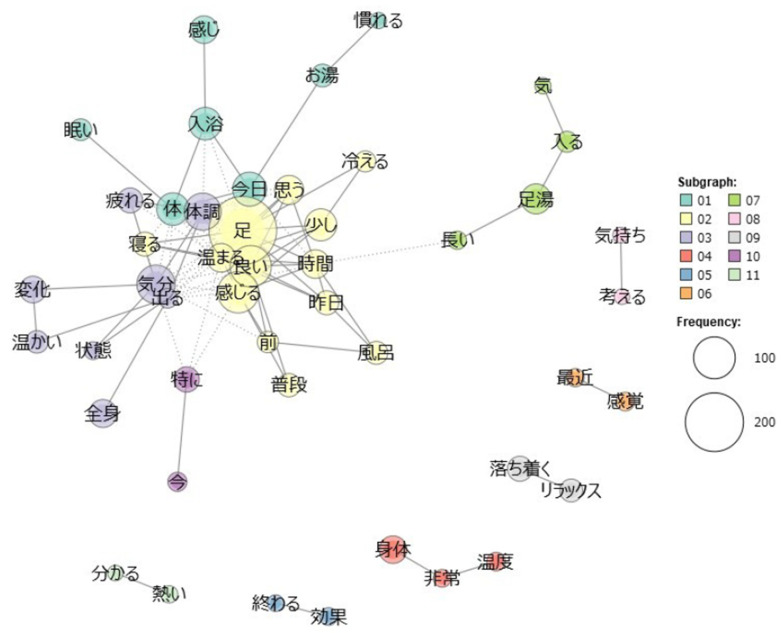
Results from a co-occurrence network analysis for the footbathing group. Japanese characters within the figure show original Japanese words extracted from the participants’ free descriptions, i.e., “温かい (warm),” “効果 (effect),” etc. See Table 3 and the main text for correspondence between each Japanese word and its English equivalent.

Results of a further co-occurrence network analysis are shown in Figure 3. In a graph of a co-occurrence network, words that are strongly related to each other are visualized with lines connecting two words. These words are classified into 11 subgraphs based on their clusters of relatedness. Words with a higher frequency of occurrence are represented by larger circular areas. The number of words drawn (nodes) was 47, the number of co-occurring relationships (edges) was 103, and the density, i.e., number of actual co-occurring relationships divided by the number of possible co-occurring relationships [44], was .095. Results revealed that *ashi* “foot” co-occurred with words such as *atatamaru* “warm up”, *yoi* “good”, *kanjiru* “feel”, and *neru* “sleep”. Words such as *kibun* “mood” and *taichou* “bodily condition” were found to co-occur with words such as *henka* “change”, *zenshin* “whole body”, and *atatakai* “warm”. In other subgraphs, words such as *kimochi−kangaeru* “feeling−think”, *saikin−kankaku* “recent−sensation”, *ochitsuku−relax* “calm−relax”, *shintai−ondo* “body−temperature”, and *owaru−kouka* “finish−effect” were associated with each other, respectively.

Coding rules for the free descriptions in the footbathing group are listed in Table 3. A total of eight codes were created, including “Somatic sensation”, “Relaxation”, “Recovery from fatigue”, “Mental health”, “Effect”, “Warming”, “Sleep quality”, and “Awareness”. After quantifying the presence or absence of a content corresponding to each code in the free descriptions, Spearman’s rank correlation coefficients between presence or absence of codes and changes in the quantitative data from pre- to post-intervention period were calculated. A statistically significant positive correlation was found between “Relaxation” and the attention regulation subscale from the MAIA (*r_s_* = .539, *p* = .007). “Effect” showed significant positive correlations with changes in scores for the observation subscale of the FFMQ (*r_s_* = .418, *p* = .042), and the SWBS (*r_s_* = .465, *p* = .022). “Warmth” significantly positively correlated with changes in deep body temperature (*r_s_* = .450, *p* = .027), and significantly negatively correlated with changes in not-distracting subscale from the MAIA (*r_s_* = −.498, *p* = .013). “Awareness” showed significant positive correlations with changes in two of the FFMQ subscale scores, describing (*r_s_* = .428, *p* = .037) and acting with awareness (*r_s_* = .438, *p* = .032), and a significant negative correlation with not-distracting subscale from the MAIA (*r_s_* = −.442, *p* = .031). Other qualitative variables did not show significant correlations with changes in scores from quantitative variables. These results show that participants who were aware of the thermogenic effects during footbathing self-reported a subjective impression of increase in the baseline of deep body temperature, and participants who reported heightened awareness of their own mind-body experience during intervention reported increased awareness of their moment-to-moment actions and interoception, and a tendency to verbalize their own actions.

## 4. Discussion

The present pilot study examined the potential physiological, endocrine, and psychological effects of footbathing in healthy university students through a 2-week daily intervention. For a large part of the measures, no statistically significant changes were observed after the intervention in laboratory assessments. Some significant changes in psychological status were observed regardless of groups, such that dispositional mindfulness and interoceptive awareness significantly increased, and trait anxiety significantly decreased, after the intervention period. However, alterations in awareness of bodily and mental states by footbathing described in participants’ daily free descriptions were significantly associated with increase in quantitative variables such as deep body temperature, dispositional mindfulness, interoceptive awareness, and subjective well-being.

### 4.1. Changes in Physiological, Endocrine, and Psychological Measures

Some of the psychological measures significantly changed regardless of the groups. Dispositional mindfulness and its some components as well as some dimensions of interoceptive awareness significantly increased, and trait anxiety significantly decreased, following the intervention period. One possible explanation for these results is that the free descriptions assigned to both groups during the intervention period increased awareness of physical and mental states [47]. Expressive writing, a practice that involves writing down one’s thoughts and emotions, is suggested as a standalone intervention aimed at enhancing physical and mental health [48]. Expressive writing is considered to alleviate negative thoughts and emotions by fostering sustained attention, re-experiencing past aversive events, and contributing to cognitive restructuring of experience [49]. In fact, previous research has shown that expressive writing about everyday life experiences and emotions can reduce anxiety [48]. Moreover, expressive writing has been reported to induce changes in autonomic nervous system activities such as lowered blood pressure, which can enhance awareness of somatic sensations [19,48]. Future research could examine effects of an intervention by footbathing that does not involve free description tasks. An alternative approach could be to use online logs or to require participants to report photos or videos of immersion during footbathing at home. These approaches may promisingly be more reliable methods to verify whether participants have adhered to the assigned intervention and prevent confounding.

From pre-intervention to post-intervention/follow-up periods, no statistically significant changes in physiological or endocrine measures were observed. These negative results might result from the relatively short intervention period. Adaptation to stimuli generally requires repetitive exposure. Meta-analyses on interventions by pilates suggest that achieving significant desirable changes in physiological health measures through physical activity typically requires 1–6 months and 50–60-min sessions two or three times per week for 24–36 sessions [50,51]. Although the content and schedules of these interventions by pilates differ from those in our study, these findings suggest that a sustained physical intervention for a longer period is necessary for physiological changes to manifest. This principle may also apply to interventions by footbathing. Indeed, McCullough et al. [11] observed a significant decrease in salivary endorphin levels after a 6-week intervention by footbathing. Therefore, a 2-week intervention may have been insufficient to induce observable changes in physiological status and endocrinological stress responses. Extended periods of footbathing may potentially result in changes in physiological/endocrinological conditions at resting states.

### 4.2. Free Descriptions During Intervention by Footbathing

A correspondence analysis revealed characteristic words within the free descriptions for each group. Participants in the footbathing group tended to describe changes in mind-body status associated with practice of footbathing, whereas those in the normal bathing group tended to describe daily life events and emotions. Additionally, a co-occurrence network analysis of the footbathing group’s descriptions primarily indicated references to changes in physical condition, mood, somatic sensations, and evaluations of footbathing. These results suggest that individuals were aware of temporary changes in psychophysiological states at least immediately after footbathing. Furthermore, the contents of descriptions from the footbathing group were associated with some quantitative variables. For example, relaxation was related to an increase in interoceptive awareness. Perception of effects of footbathing was associated with increases in mindfulness and subjective well-being. Awareness of the warming effect corresponded to an increase in the deep body temperature. Moreover, a heightened awareness during the intervention period was associated with increases in interoceptive awareness and dimensions of mindfulness including describing and acting with awareness. These results suggest that awareness of changes in mental and bodily status associated with footbathing may contribute to improvements of actual psychophysiological status. These results are consistent with several lines of studies supporting the notion that an intervention which provides interoceptive stimuli promotes self-awareness of one’s physical and mental states [19,20,21]. Future studies should examine effects of footbathing on quantitative indices of interoceptive awareness and physical and mental health with longer intervention periods and larger sample sizes.

### 4.3. Limitations and Future Perspectives

The present study has several major limitations. First, a 2-week intervention may not be long enough to yield significant changes in most physiological, endocrine, and psychological measures. Previous studies have shown that interventions by footbathing combined with aromatherapy lasting for six or eight weeks lead to physiological, endocrinological, and psychological changes [11,12]. In physical interventions, physiological adaptation to a stimulus is suggested to typically require a period of 1 to 6 months [50]. In the present study, participants’ self-reports following daily footbathing were suggested to involve short-term warming effects, increased awareness of somatic sensations, and improved mood states. A limited number of sessions in the present study may have resulted in temporary changes in mental and physical states immediately after the practice, but these effects likely disappeared over time, preventing long-term plastic changes in the mind and body. Therefore, a longer intervention by footbathing, e.g., six or eight weeks, may potentially lead to significant changes in physiological and endocrinological activities at resting state as well as psychological traits.

Second, methodology of footbathing has not been sufficiently established. To date, the amount of research on the effects of footbathing is limited, and procedures for intervention are not consistent. Regarding water levels, both the present and previous studies have primarily specified a depth below the knees or ankles. Some previous studies estimated the optimal water levels for footbathing. Fujihira and Nakamura [52] suggested that footbathing at 40 °C for 10 min immersing the feet up to the ankles was more effective for warmth retention in adult men. Shimizu and Nagatani [53] examined changes in mood status of healthy adult women after footbathing at 41 °C for 15 min, and found that the water level of 15 cm more greatly improved mood compared to the depths of 8 cm and 20 cm. These findings suggest that footbathing while immersing the feet up to the ankles may yield greater psychophysiological effects than footbathing while covering the entire lower legs. One possible explanation is that immersing the entire lower legs in hot water could expand blood vessels in the gastrocnemius muscle, which pumps blood back to the heart. The muscle warming might lessen the pump effect, resulting in blood pooling in the lower limbs and impairing blood circulation in the whole body [53]. In the present study, participants were instructed to immerse their entire lower legs, which may have stagnated blood circulation in a similar way. This may have led to non-significant changes observed in baseline physiological, endocrine, or psychological traits by the footbathing intervention. However, these preceding studies involved different water temperatures, durations of footbathing, demographics of participants, etc. Given these various conditions, the ideal water temperature and interventions lasting for various lengths need to be further investigated. Moreover, since the intervention was conducted at home, it may be necessary to use online logs or videos/photos of foot immersion to verify whether each participant’s footbath practice was performed by adhering to the instructions.

Third, the generalizability of the results to other populations should be examined. Although previous studies have shown effects of footbathing at the physiological, endocrine, and psychological levels, these effects may vary with age. For example, Miwa et al. [54] reported a difference in autonomic nervous functions including deep body temperature, heart rate, skin blood flow, and blood pressure between young and older adults after footbathing for 20 min. Younger adults exhibited greater increases in deep body temperature, heart rate, and blood flow as compared to older adults. Blood pressure declined significantly in older adults but did not in young adults. Intervention studies involving footbathing lasting for several weeks should investigate whether similar trends are observed when comparing different age groups. However, older adults are suggested to have lower interoceptive awareness and are less likely to experience psychophysiological changes compared to younger adults [54,55]. Therefore, older adults may require a longer intervention period for footbathing to achieve noticeable mind-body changes. Furthermore, research on footbathing including the present study to date has involved relatively homogenous samples. Potential benefits of footbathing as a regimen would apply not only to university students but also to a wide range of age groups such as adolescents and elderly people. Therefore, future research may investigate the effects of footbathing in a broader age demographic, spanning from young to older adults. In addition, applicability of footbathing for various psychosomatic disorders should further be investigated. Certain psychosomatic disorders may be associated with reduced meta-awareness of interoception or irregular endocrine and physiological activities. For example, these mechanisms are considered to underlie irritable bowel syndrome (IBS), which is characterized by symptoms such as abdominal discomfort and bowel movement disturbances [56]. IBS patients are thought to exhibit hypersensitivity and unreliability in their interoception, suggesting inaccurate representations of interoceptive signals [57,58]. In the present study, awareness of interoception indicated in the participants’ free descriptions were suggested to be associated with improvements in physiological, endocrine, and psychological health outcomes. Interventions aimed at enhancing interoceptive meta-awareness are believed to improve accuracy in representations of interoception, promoting moment-to-moment adaptive responses and homeostatic regulation of bodily states [19,20,21,59,60]. Thus, footbathing interventions may potentially alleviate physical symptoms by modulating underlying neural mechanisms underlying psychosomatic disorders such as IBS. These issues should require further investigations in the future quest.

## 5. Conclusions

The present pilot study did not reveal statistically significant changes by footbathing in quantitative variables through the intervention period. However, their associations with qualitative data suggested that awareness of changes in psychophysiological states may be involved in process of enhancing the baseline of body temperature, dispositional mindfulness, interoceptive awareness, and well-being by footbathing. The intervention will be feasible with modification of methodology, such as extension of intervention period, abolishment of free descriptions, establishment of procedures, and larger sample size for a main study.

## Figures and Tables

**Table 1:** Descriptive statistics for the total scores and subscales from each scale.

Group	Footbathing M (*SD*)			Normal Bathing M (*SD*)		
Measurement Period	Pre-	Post-	Follow-Up	Pre-	Post-	Follow-Up
Deep body temperature	36.84 (0.39)	36.88 (0.36)	36.91 (0.32)	36.86 (0.34)	36.88 (0.42)	36.93 (0.37)
Systolic blood pressure	105.98 (10.45)	107.77 (9.50)	108.54 (8.31)	108.88 (10.52)	108.14 (11.95)	108.17 (9.26)
Diastolic blood pressure	66.38 (8.29)	66.15 (8.46)	67.46 (6.22)	69.41 (8.44)	66.74 (8.51)	66.43 (7.38)
Cortisol (μg/dL)	0.201 (0.102)	0.183 (0.110)	0.203 (0.105)	0.196 (0.099)	0.213 (0.132)	0.183 (0.092)
FFMQ total	124.04 (15.29)	128.58 (15.79)	130.75 (18.43)	123.52 (14.49)	126.52 (16.14)	127.33 (16.74)
Observing	26.54 (4.94)	27.33 (4.40)	27.63 (6.79)	25.52 (5.68)	26.67 (5.63)	26.81 (5.58)
Non-reactivity	21.04 (2.87)	21.88 (3.43)	22.21 (3.88)	21.68 (4.67)	22.38 (3.94)	23.00 (3.42)
Non-judging	25.38 (6.67)	27.75 (6.12)	28.75 (6.25)	24.86 (4.95)	25.43 (4.87)	25.33 (6.95)
Describing	26.04 (6.79)	26.33 (6.34)	26.75 (7.03)	27.29 (5.78)	27.76 (5.90)	27.52 (6.91)
Acting with Awareness	25.00 (5.23)	25.33 (4.84)	25.42 (5.55)	24.52 (5.62)	23.95 (5.08)	24.67 (6.05)
MAIA	―	―	―	―	―	―
Attention Regulation	3.44 (0.88)	3.57 (0.88)	3.76 (0.99)	3.43 (0.80)	3.68 (0.80)	3.69 (0.99)
Body Listening	3.12 (1.20)	3.67 (1.03)	3.70 (1.50)	3.41 (1.20)	3.64 (1.33)	3.58 (1.20)
Noticing	3.85 (0.86)	3.86 (0.82)	4.07 (1.09)	3.78 (0.87)	4.06 (0.84)	3.90 (1.18)
Emotional Awareness	4.10 (1.14)	4.28 (1.22)	4.47 (1.16)	4.02 (1.36)	4.14 (1.20)	4.38 (1.19)
Trusting	3.89 (1.39)	4.14 (1.13)	4.13 (1.07)	3.67 (1.16)	4.10 (1.16)	4.51 (1.07)
Not-distracting	3.54 (1.37)	3.25 (1.16)	3.29 (1.16)	3.52 (1.37)	3.81 (1.07)	3.79 (1.09)
SWBS	44.36 (6.45)	45.79 (7.03)	45.38 (7.06)	45.76 (6.43)	45.95 (6.12)	45.62 (6.51)
STAI-T	46.46 (9.90)	44.17 (8.89)	43.92 (9.17)	45.95 (10.61)	45.48 (10.18)	43.57 (10.79)
CES-D	13.50 (7.30)	11.73 (7.32)	12.63 (9.38)	12.24 (6.59)	13.29 (7.87)	10.24 (6.59)

**Table 2:** Results from two-way ANOVAs with group (footbathing/normal bathing) and measurement period (pre-/post-/follow-up).

Measures	Main Effects (Group)				Main Effects (Measurement Period)				Interactions (Group × Period)			
	*df*	*F*	*p*	*ηp^2^*	*df*	*F*	*p*	*ηp* ^2^	*df*	*F*	*p*	*ηp* ^2^
Deep body temperature	1, 43	0.026	.873	<0.001	2, 86	1.372	.259	0.031	2, 86	0.066	.936	0.002
Systolic blood pressure		0.122	.729	0.003	2, 86	0.434	.649	0.010	2, 86	1.489	.231	0.033
Diastolic blood pressure		0.198	.658	0.005	2, 86	0.790	.457	0.018	2, 86	1.525	.223	0.034
Cortisol (μg/dL)		0.004	.951	<0.001	2, 86	0.054	.947	0.0013	2, 86	1.043	.357	0.024
FFMQ total		0.193	.663	0.004	2, 86	6.983	.002 **	0.140	2, 86	0.500	.608	0.011
Observing		0.318	.576	0.007	2, 86	1.885	.158	0.042	2, 86	0.037	.964	<0.001
Non-reactivity		0.432	.514	0.010	2, 86	4.968	.009 **	0.104	2, 86	0.064	.938	0.0015
Non-judging		1.625	.209	0.036	1.737, 74.675	4.622	.017 *	0.097	1.737, 74.675	2.442	.101	0.054
Describing		0.377	.542	0.009	2, 86	0.625	.538	0.014	2, 86	0.282	.755	0.007
Acting with Awareness		0.334	.566	0.008	1.558, 67.012	0.327	.668	0.008	1.558, 67.012	0.420	.608	0.010
MAIA	―	―	―	―	―	―	―	―	―	―	―	―
Attention Regulation	1, 43	0.000	.983	<0.001	2, 86	2.835	.064	0.062	2, 86	0.376	.688	0.009
Body Listening		0.029	.866	0.001	2, 86	5.130	.008 **	0.107	2, 86	1.152	.321	0.026
Noticing		0.003	.956	<0.001	2, 86	1.115	.333	0.025	2, 86	1.269	.286	0.029
Emotional Awareness		0.097	.757	0.002	1.573, 67.624	2.607	.093	0.057	1.573, 67.624	0.024	.953	<0.001
Trusting		0.018	.894	<0.001	1.498, 64.402	5.382	.013 *	0.111	1.498, 64.402	1.754	.188	0.039
Not-distracting		1.275	.265	0.029	1.602, 68.888	0.052	.918	0.0012	1.602, 68.888	1.428	.246	0.032
SWBS		0.098	.755	0.002	1.500, 64.520	1.448	.241	0.033	1.500, 64.520	1.051	.338	0.024
STAI-T		0.003	.957	<0.001	1.550, 66.631	5.714	.009 **	0.117	1.550, 66.631	0.948	.372	0.022
CES-D		0.059	.810	0.0014	2, 86	1.424	.246	0.032	2, 86	3.858	.025 *	0.082

*: *p* < .05; **: *p* < .01.

**Table 3:** Codes and coding rules for free descriptions in the footbathing group.

Codes (*N*)	Coding Rules
Somatic sensation (23)	*shintai/karada/zenshin/jyohanshin* “body”, *chi* “blood”, *kekkou* “blood circulation”, *kesshoku* “complexion”, *ashi* “foot” + *karui* “light”, *shinzou* “heart”, *tainai* “within the body”, *taichou* “physical condition”, *hihu* “skin”, *hyoumen* “surface”, *ichou* “gut”
Relaxation (16)	“relax”, *ochituku* “calm down”, *odayaka* “calm”, *kokochiyoi/kimochiyoi* “feeling good”, *yasuragi/yasuragu* “peace”
Recovery from fatigue (23)	(*tsukare/hirou* “fatigue” or *itami* “pain”) + (*keigen* “alleviation” or *nai* “no/not” or *toreru/kaishou/kaihuku* “recovery”), “reflesh”
Mental health (18)	((*kibun* “mood” or *seishin/kokoro/kimochi* “mind” or “mental”) + (*yoi/ryoukou* “good” or *antei* “stabilization”)), positive, *ureshii* “glad”, *tanoshii/tanoshimeru* “enjoyable”
Effects (14)	*kouka* “effect”, *yukou* “effectiveness”, *eikyou* “impact”, *henka* “change”, (+ nai/kanjinai were excluded)
Warming (21)	*atatamaru* “getting warm”, *atatakai* “warm”, *atsui* “hot”, *pokapoka* “feel comfortably warm”, *ase* “perspiration”, *hakkan/asebamu* “perspire”, *hoon* “retain heat”
Sleep Quality (21)	*suimin/nemuri* “sleep”, *nemui/nemutai* “sleepy”, *nemureru* “can easily sleep”, *netsuki/netsuku/shushin/nyumin* “fall asleep”, mezame “awaking” (+ *warui* “bad” or *osoi* “slow” were excluded)
Awareness (22)	*kankaku* “sensation”, *wakaru* “understand”, *ishiki* “contiousness”, *kanjiru* “feel”, *kanji* “feeling”, *kiduku/(kankaku* + *ki)* “notice”, *binkan/kabin* “sensitive”

## Data Availability

The datasets used and/or analyzed during the study are available from the corresponding author upon reasonable request.
